# Exploring the employment determinants of job insecurity in the French working population: Evidence from national survey data

**DOI:** 10.1371/journal.pone.0287229

**Published:** 2023-06-14

**Authors:** Isabelle Niedhammer, Elodie Pineau, Sandrine Bertrais, Duncan Gallie

**Affiliations:** 1 Irset (Institut de Recherche en Sante, Environnement et Travail)—UMR_S 1085, INSERM, University Angers, University Rennes, EHESP, ESTER Team, Angers, France; 2 Nuffield College, University of Oxford, Oxford, United Kingdom; St John’s University, UNITED STATES

## Abstract

Studies are lacking on the employment determinants of job insecurity, that may be helpful to determine highly exposed groups and to assess the feasibility of constructing job-exposure matrices (JEMs) for this occupational exposure. The objectives were to explore the employment determinants of job insecurity in a nationally representative sample of the French working population. The study was based on the cross-sectional data of the 2013 national French working conditions survey including a sample of 28,293 employees, 12,283 men and 16,010 women. Job insecurity was assessed using one single item related to the fear of job loss in the next 12 months. Gender, age, and educational level were studied as well as the following employment variables: temporary/permanent work contract, full/part time work, job seniority, occupation, economic activity of the company, public/private sector, and company size. The associations with job insecurity were studied using bivariate and multivariate analyses. One quarter of the study sample was exposed to job insecurity, without any difference between genders. Lower age and lower educational levels were associated with job insecurity. Employees who had a temporary work contract, lower job seniority, who were working in low-skilled occupational groups, in manufacturing (for both genders) and construction (among men), and in the private sector had a higher prevalence of exposure to job insecurity. The two major employment variables associated with job insecurity were temporary work contract (prevalence ratios>2) and private sector (prevalence ratios>1.4) for the whole sample and for both men and women. Our findings suggested that intervention/prevention measures could be oriented towards specific highly exposed groups of the working population, especially those exposed to temporary work contract and/or working in the private sector. Our study also underlined that constructing JEMs for job insecurity may be possible and could be a useful tool for large-scale occupational health studies.

## Introduction

Job insecurity, defined as the fear of job loss, is considered as a major psychosocial job stressor, because work has a central role in people’s life, because job security is a crucial aspect of the “psychological contract” between employee and employer [1], and because job insecurity may also refer to the potential risk of unemployment and its detrimental consequences. Job insecurity was found as a risk factor for various health outcomes [2], including coronary heart diseases [3], diabetes [4], and mental health outcomes such as depression, anxiety, burnout, suicide ideation, and psychotropic medication use [5–8]. The exposure to job insecurity can be as harmful as the exposure to unemployment for mental health outcomes [6,9], and was even more strongly associated with somatic symptoms than unemployment, whereas unemployment displayed stronger associations with general health status and mortality than job insecurity [9]. Whereas the literature has been abundant on the effects of job insecurity, studies have been less frequent on its determinants [10]. This underlines the need for more research on job insecurity and its determinants, which may be of great interest in a preventive point of view. Determinants of job insecurity may be grouped into the following groups [11]: country-level or macroeconomic factors (unemployment, economic crisis, regulations, etc.), company-level or organisational factors (changes, systems, norms, culture, policies, etc.) and individual factors (demographics, employment characteristics, personality, etc.). A literature review summarized the role of labour market and social protection welfare institutions and policies in job insecurity [12] and some recent studies explored this topic extensively [13,14]. Despite the harmful effects of job insecurity on health, the literature on the employment determinants of job insecurity is relatively more seldom, especially in the case of France. The identification of employment determinants of job insecurity is therefore likely to be helpful to shape policies oriented towards highly exposed groups in working populations.

Four literature reviews [1,10,15,16] and two overviews [12,17] explored the employment determinants of job insecurity specifically or among other determinants. Employment characteristics have been defined by Gallie et al. [18] by characteristics related to “contractual status and class position”. These characteristics may cover various employment-related variables that may influence job insecurity and may also be helpful to identify the groups particularly exposed to job insecurity and thus the targets of preventive measures.

In addition, job-exposure matrix (JEM), a well-known tool in the assessment of occupational exposures, may be planned in the case of identified employment determinants that can serve as job title variables in the construction of such JEMs. Indeed, a JEM is a matrix in which job titles are presented in lines and exposures in columns, and exposure estimate in terms of frequency, intensity of exposure, etc. in the intersection of each line and column. Occupation is a very commonly used job title variable in the construction of JEMs. Economic activity of the company was also used in addition to occupation in some studies, but using more than one job title variable remains seldom in the construction of JEMs. However, previous studies suggested that the use of several job title variables simultaneously may be useful to construct JEMs for psychosocial work exposures [19]. The identification of the relevant job title variables, i.e. relevant employment determinants, is thus fundamental. Indeed, identifying these employment variables would allow to construct JEMs that could be used and applied for large-scale studies in which no data about exposure has been collected, but only employment variables.

Previous reviews/overviews and previous studies explored one or several employment determinants of job insecurity and showed that the type of work contract (temporary/permanent), full/part time work, job seniority, occupation, economic activity of the company, public/private sector, or company size might be associated with job insecurity [1,10,12,13,15–18,20–32]. Nevertheless, each of these reviews or studies often focused on a very limited number of employment variables. There was thus a need to provide more information on the employment determinants of job insecurity.

France was found as one of the countries with the highest level of job insecurity in Europe and in the OECD countries [26,33], although this was not the case in the most recent years [34,35]. Furthermore, studies showed that the determinants of job insecurity may be country-specific [1,30,31]. Thus, the study of the determinants of job insecurity in France should be informative not only for this given country but also to better understand the differences between countries.

The objectives of the study were to explore the associations between employment variables and job insecurity in the national French working population of employees, to determine highly exposed groups, and to evaluate whether the construction of a JEM would be possible for this exposure. The study contributed to the literature in a variety of ways. The large nationally representative sample, that was used, allowed the results to be generalizable to the national working population. We were able to study a large set of employment variables at the same time and not only one. Attention was paid to gender differences and the respective situation of men and women on this topic. Thus, we were able to fulfil these objectives in order to provide information that may be useful for occupational health prevention policies.

## Materials and methods

### Study sample

The study relied on the data from the 2013 national French working conditions survey that was set up by the DARES of the French ministry of labour. This survey included a sample of 29,556 employees aged 15–65 years old, that was nationally representative of the French working population of employees and obtained through a two-stage random sampling design. First, households were selected randomly from the 2011 French census, and then workers were selected randomly within each selected household, in which there were more than one worker. The data were collected using a questionnaire asked by interviewers. Our study was thus a quantitative study. The survey was approved by French ethics committees (CNIL no 2012–288 and CNIS no 2010-245/D130). Written consent was obtained and for minors consent was obtained from parents. All data were fully anonymized before access.

### Job insecurity

Job insecurity was assessed using one binary item that was related to the fear of job loss in the next 12 months.

### Employment variables

The following variables were explored in association with job insecurity. The variables selected were derived in a modified form from the broad classification used by Böckerman [23]. This captures the fact that job insecurity may be affected by two types of employment variables: the characteristics of the specific job held by the individual and the characteristics of the wider organisation or company in which the job is situated. With respect to job characteristics, it may be affected by the degree of formal protection associated with the contractual status of the post, by differences in job specific human capital and employment norms relating to length of service, and by differences between employees with different levels and types of skill in exposure to change, employability and replaceability. With respect to company characteristics, job security arguably may be affected by the economic activity of the company (with its implications for the volatility of the market for its products or services), differences in employment norms associated with different types of ownership, and differences in human resource policies and capacities linked to organisational size.

The variables we retained were:

1) Job characteristics
  - temporary/permanent work contract
  - full/part time work (part time work was defined by any working time lower than full time, i.e. lower than 35 hours a week which has been the norm in France since 2000)
  - job seniority (≤1, 1–5, 5–10, or >10 years)
  - 3 variables for occupation, coded using the French classification [36], were related to different levels of the classification and included 4, 14 and 25 groups respectively.
2) Company characteristics
  - 3 variables for economic activity of the company, coded using the French classification [37], were related to different levels of the classification and included 4, 17 and 38 groups respectively.
  - public/private sector of the company
  - company size (total number of employees): small (<50 employees), medium (50–499 employees) and large (500 or more employees)

### Covariates

We studied gender, age, and educational level.

### Other variables

While our main concern was with employment variables that may have general effects for workers in specific employment positions, there may be other aspects of employment experience that may affect the job security of individual workers. The following variables were studied in sensitivity analyses and included:

- trade union membership
- availability of sufficient and appropriate in-work training
- exposure to technical changes within the last 12 months
- period of unemployment for one year or more in the past
- non-working period of one year or more due to health-related problems in the past

### Statistical analyses

All the analyses used weighted data to take non-response and marginal calibration into account. All our analyses were also performed following high standards for gender research [38]. Indeed, it was legitimate to consider that there might be gender differences, whether deriving from discrimination, role constraints or choice, in the prevalence of the studied variables (job insecurity, employment variables, covariates, etc.) and in the associations between the studied variables and job insecurity. For this reason, we tested these gender differences systematically.

A description of the sample for all variables was done for the whole sample and for men and women separately. Comparison between genders was performed using the Rao-Scott Chi-2 test, which is a corrected version of the Pearson Chi-2 test for complex surveys with weighted data. Firstly, the associations of gender, age, educational level, and employment variables with job insecurity were tested using the Rao-Scott Chi-2 test. Secondly, these associations were studied using Poisson regression models with robust variance estimation and gender-related interactions were tested. In the case of significant gender-related interactions, we presented the results for men and women separately, if not we presented the results for the whole sample. Prevalence estimates and 95% confidence intervals were presented using forest plots.

The following sensitivity analyses were performed: (1) robust Poisson regression models with additional adjustment for age were performed for the study of the associations between each employment variable and job insecurity, (2) robust Poisson regression models including all employment variables simultaneously were performed in association with job insecurity, (3) forward stepwise robust Poisson regression models (p-value<0.05 as criterion to entry into the model) were performed, (4) the analyses were redone after classifying employees who did not respond to the item of job insecurity (4.4%) as non-exposed, indeed, most of these non-respondents did not know whether they might fear losing their job, which might be interpreted as the absence of actual threat of job loss, (5) the analyses were performed again using job insecurity combined with difficulty of finding equivalent job, and (6) we studied the associations between the other variables and job insecurity and we examined whether each of these variables changed the associations between employment variables and job insecurity.

We used R software for all statistical analyses, as R allowed us to perform all these analyses using weights.

## Results

### Description of the study sample

Among the sample of 29,556 employees, 1,263 employees (i.e. 4.4%) had missing values for the item of job insecurity. Consequently, the study sample included 28,293 employees, 12,283 men and 16,010 women. The prevalence of exposure to job insecurity was found to be 25.2% (95% CI: 24.3–26.1) in the study sample, without any significant difference between genders. The description of all studied variables is presented in S1 Table. Significant differences between genders were observed for age, educational level, full/part time work, occupation, economic activity of the company, public/private sector, and company size.

### Associations between covariates, employment variables and job insecurity

**Fig 1:**
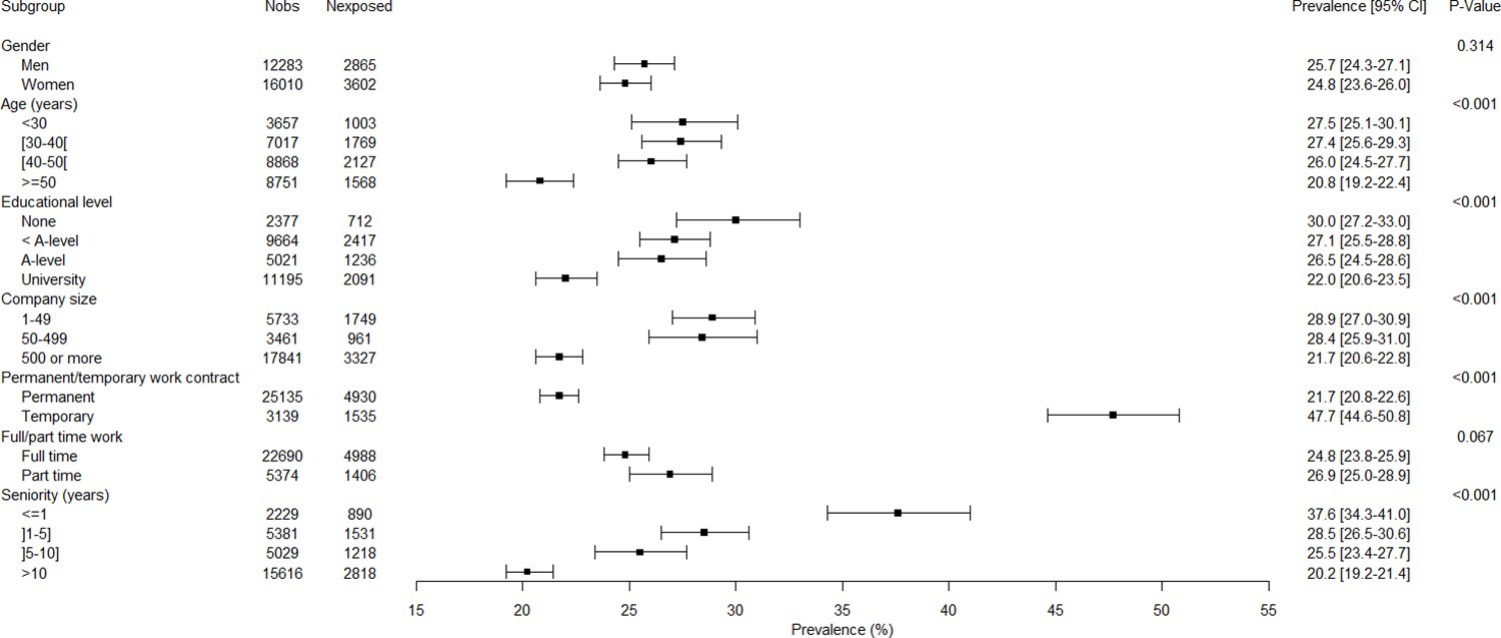
Weighted prevalence of exposure to job insecurity according to gender, age, education level, company size, work contract, full/part time work and seniority among the study sample.

**Fig 2:**
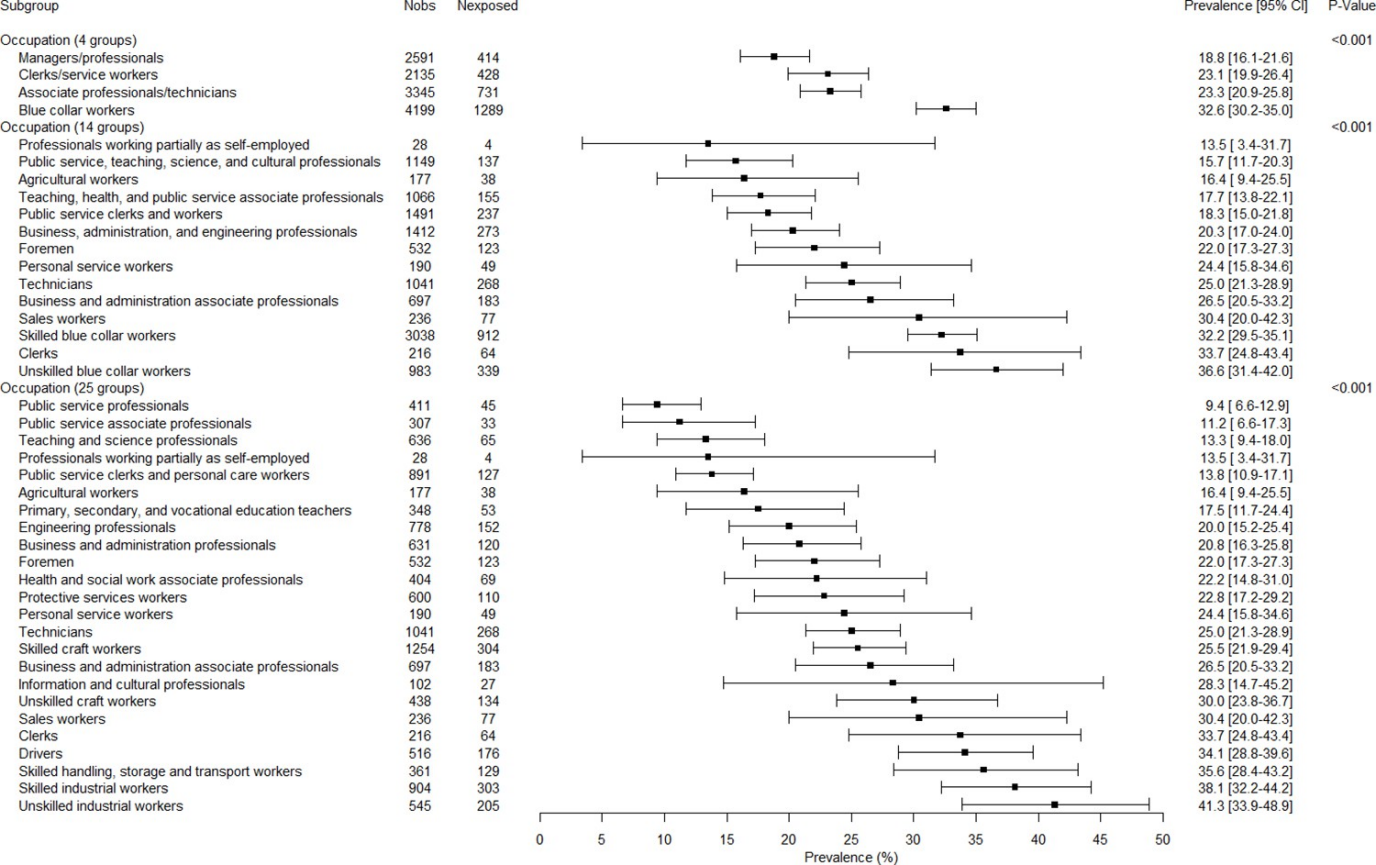
Weighted prevalence of exposure to job insecurity according to occupation among men.

**Fig 3:**
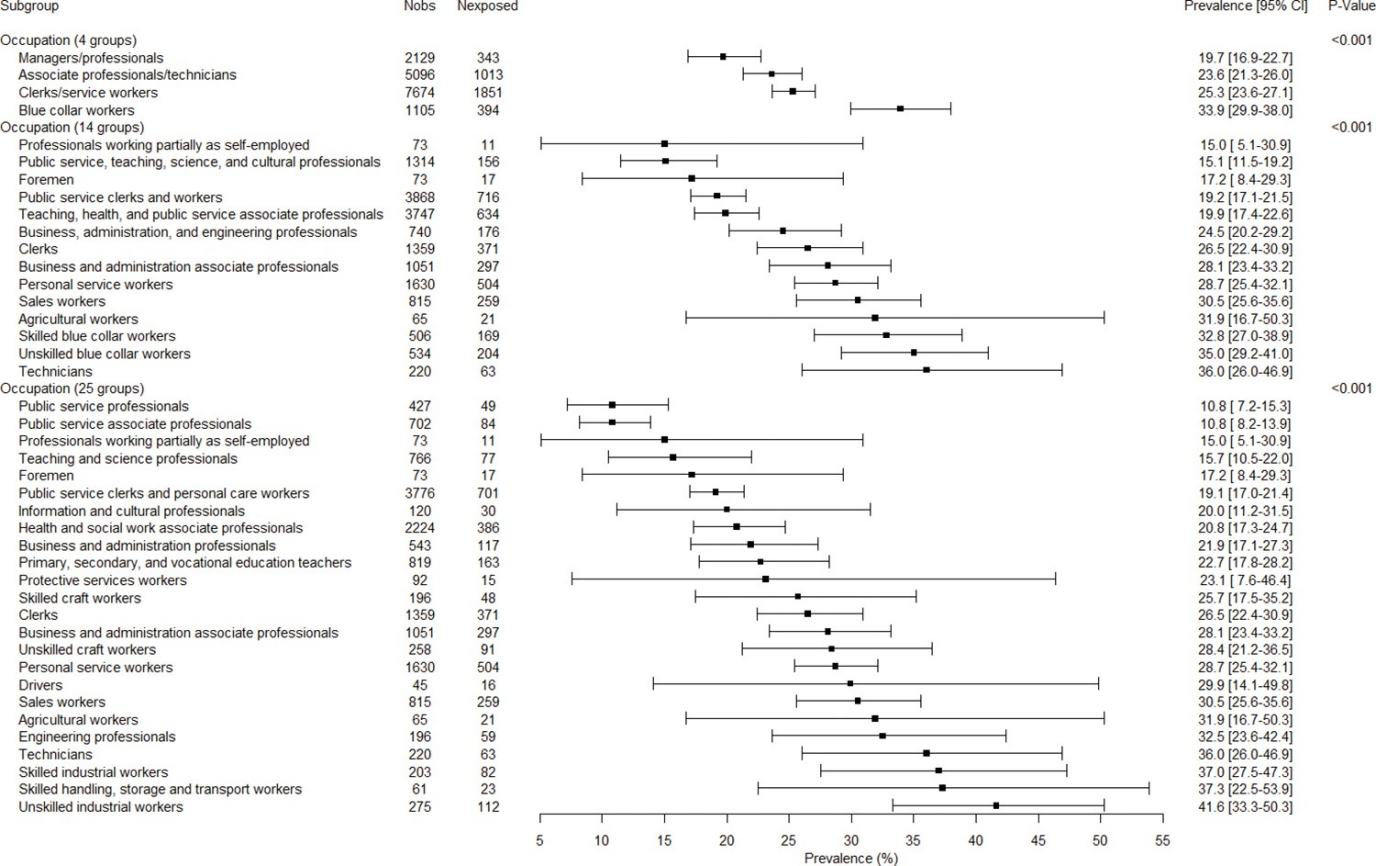
Weighted prevalence of exposure to job insecurity according to occupation among women.

**Fig 4:**
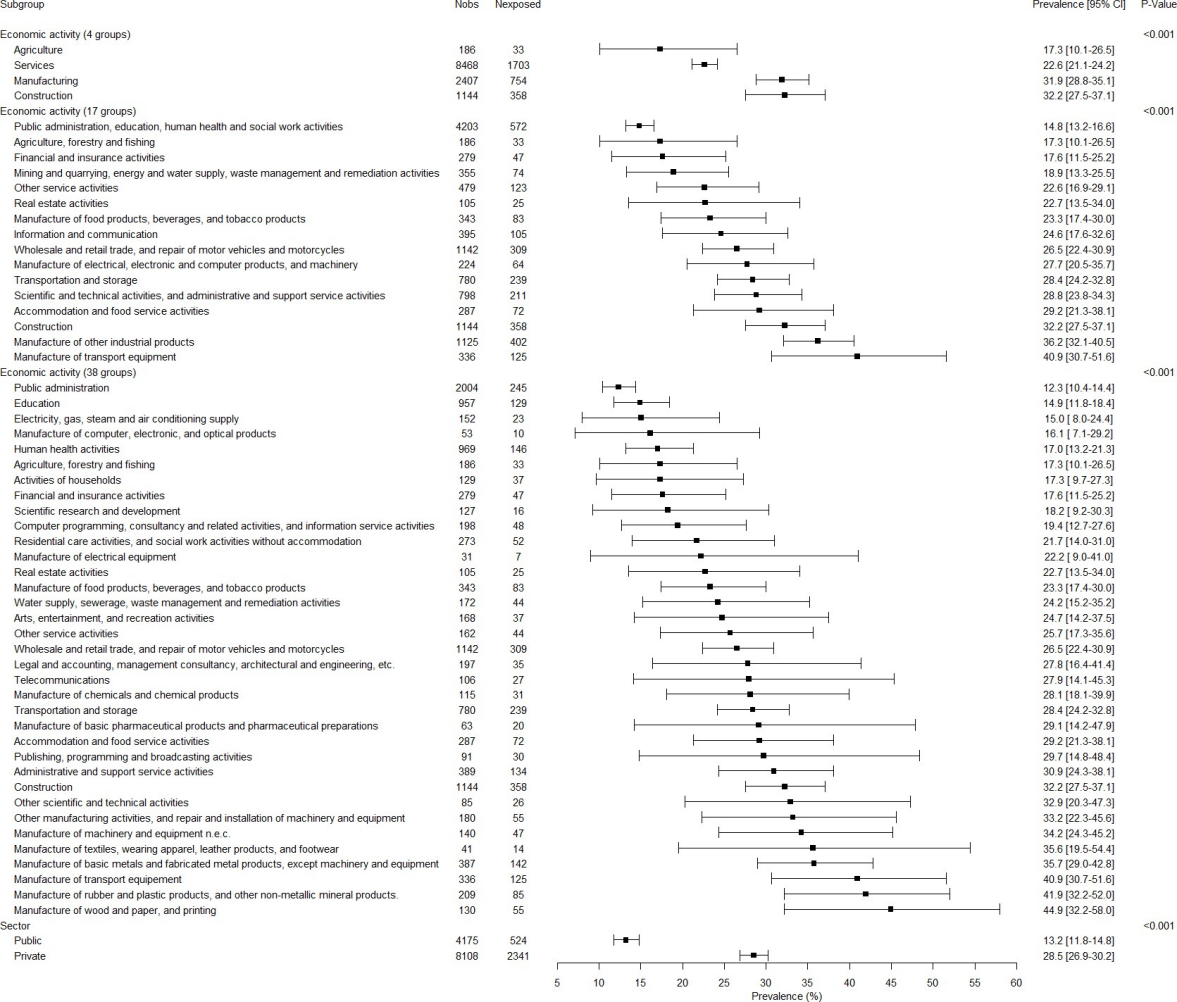
Weighted prevalence of exposure to job insecurity according to economic activity of the company and public/private sector among men.

**Fig 5:**
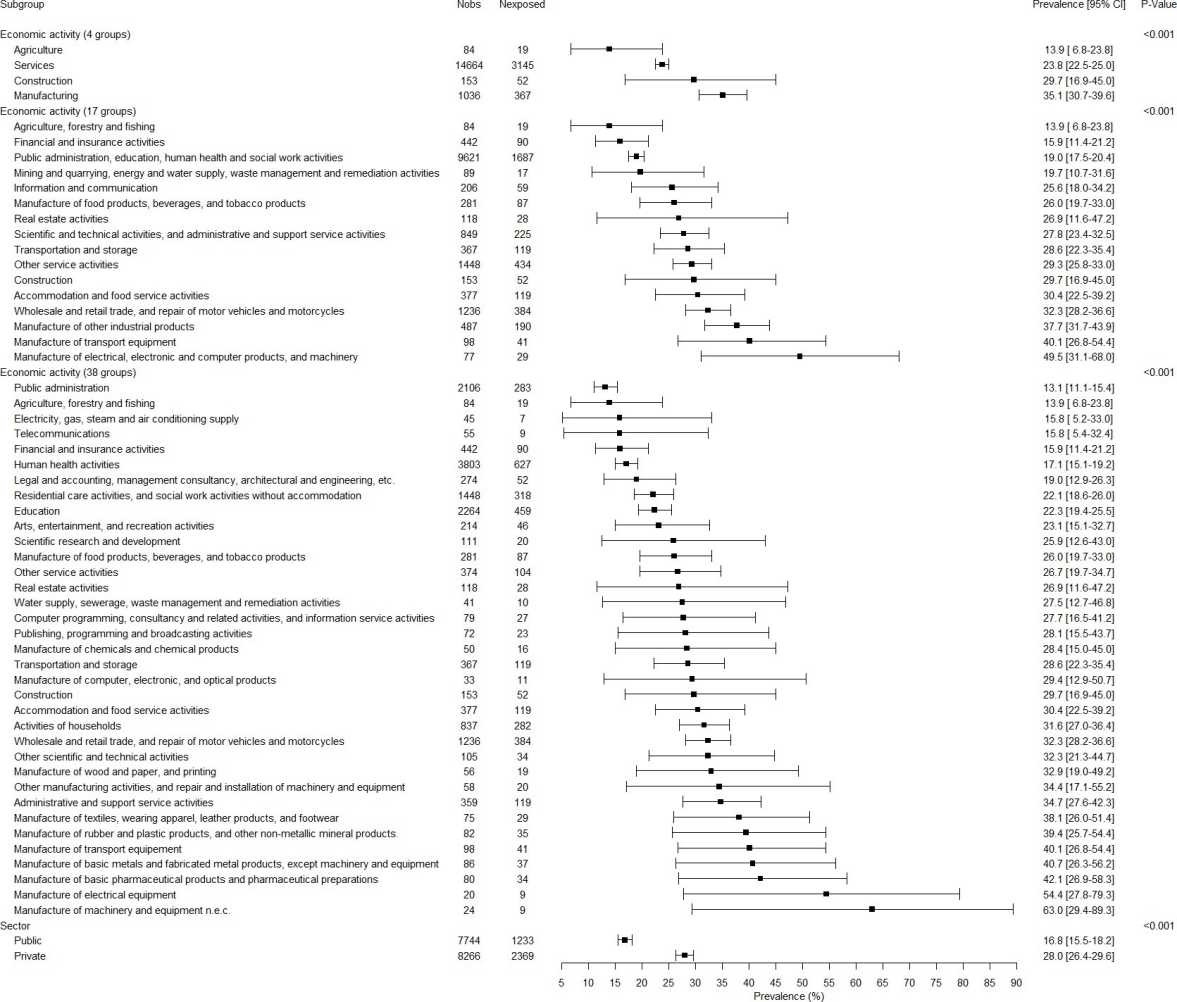
Weighted prevalence of exposure to job insecurity according to economic activity of the company and public/private sector among women.

The associations between all variables and job insecurity are presented in Figs 1–5. There were no gender-related interactions for age, educational level, company size, work contract, full/part time work, and job seniority in association with job insecurity, and consequently the results were presented among the whole study sample (Fig 1). The prevalence of exposure to job insecurity was higher among the employees aged less than 50, among those with lower educational levels, among those working in small/medium companies, among those with a temporary work contract, and among those with lower job seniority (Fig 1). Significant gender-related interactions were found for occupation, economic activity, and public/private sector in association with job insecurity, and consequently the results were presented for men and women separately. Blue collar workers, and particularly unskilled blue collar and industrial workers, had a higher prevalence of job insecurity among both genders (Figs 2 and 3). Employees working in the manufacturing and construction (for men) and in the manufacturing (for women) had a higher prevalence of job insecurity. The highest prevalences of exposure were found in different types of manufacture (Figs 4 and 5). The prevalence of job insecurity was higher among employees working in the private sector and the gap between the public and private sector was bigger among men than among women (Figs 4 and 5).

### Sensitivity analyses

Sensitivity analysis #1 provided similar results when we adjusted for age. Some differences in the results were observed in the multivariate analyses of sensitivity analysis #2 compared to the bivariate analyses presented in Figs 1–5. The models including all variables simultaneously showed that the significance or magnitude of some associations was reduced (S2 and S3 Tables). The lower magnitude of the association between age and job insecurity was explained by the adjustment for job seniority and work contract, age being associated with these two variables. The association between educational level and job insecurity disappeared after adjustment for occupation, as there was an association between educational level and occupation. The association between company size and job insecurity was no longer significant after adjustment for public/private sector, the public sector being part of large companies. Forward stepwise models performed in sensitivity analysis #3 showed that the selected variables were for all, for men, and for women: temporary/permanent work contract, public/private sector, age, and economic activity, plus occupation (for all and for men) and job seniority (for all) (S4 and S5 Tables). The results were unchanged when we classified the employees who did not respond to the item of job insecurity as non-exposed in sensitivity analysis #4. Sensitivity analysis #5 with job insecurity combined with difficulty of finding equivalent job led to a lower prevalence of exposure (8.3%, 95% CI: 7.7–9.0) but the associations with the employment variables were very similar. The study of the other variables in sensitivity analysis #6 showed that trade union membership was not associated with job insecurity whereas no sufficient and appropriate in-work training, exposure to technical changes within the last 12 months, period of unemployment for one year or more in the past, and non-working period of one year or more due to health-related problems in the past were associated with a higher prevalence of exposure to job insecurity in the bivariate analyses. However, taking these variables into account did not change the significance or magnitude of the associations between employment variables and job insecurity substantially. Trade union membership became significantly associated with job insecurity in the multivariate analyses.

## Discussion

### Main results

Our study showed that job insecurity was an exposure for which about one quarter of the national French working population of employees was exposed. There was no difference between genders. Lower age was associated with a higher prevalence of exposure to job insecurity, echoing the association between lower job seniority and job insecurity. Lower educational levels also increased the prevalence of exposure of job insecurity, although this association disappeared after adjustment for occupation. Among the employment variables, the most important variables associated with job insecurity were temporary work contract and the private sector. There was a social gradient between occupation and job insecurity, and employees with lower occupational levels had a higher prevalence of exposure. Manufacturing for both genders, and construction for men were the sectors with the highest prevalence of exposure to job insecurity. We found no association between company size and job insecurity after adjustment for public/private sector, and no association as well between full/part time work and job insecurity. Our study suggested that the construction of JEMs for job insecurity may be possible and should use various employment variables as job title variables to increase the validity of such JEMs.

### Limitations and strengths

Our study included a number of limitations. We studied job insecurity as the fear of job loss, which is the most classical way of defining job insecurity, and did not explore other forms of job insecurity, i.e. other threats, that may face employees, to job status (threats of loss of valued job features) and that may be associated with other individual and employment variables [18]. We used cross-sectional data, consequently no causal conclusion could be drawn from our results. Nevertheless, reverse causation appeared unlikely for the observed associations. We used the term ‘determinants’, as did other authors. Some other authors used the terms ‘antecedents’ or ‘predictors’. In no case, these terms should be interpreted as causality. We studied a set of individual and employment variables in association with job insecurity, and did not pretend to cover all potential determinants of this exposure. Indeed, our study covered two objectives, i.e. the study of employment variables associated with job insecurity but also the study of the feasibility of constructing JEMs for job insecurity. Nevertheless, we were able to study other variables associated with job insecurity for which data were available and may be informative for a wider scope of the determinants of job insecurity. The national working conditions survey data collected in 2013 were used, and it would be informative to study whether our results can be replicated over time and to test whether changes might have occurred over time in France. Indeed, other studies showed that the determinants of job insecurity, in particular occupation, had changed over time in the UK [18]. Furthermore, it is likely that the Covid pandemic may have had an influence on job insecurity and its determinants; this point could not be assessed given no data of the national French working conditions survey were available after the pandemic. This may be particularly important as regards the construction of JEMs for job insecurity, for which a time dimension might be useful if the employment variables were not consistently associated with job insecurity over time.

The following strengths of our study should be underlined. The study was based on a large nationally representative sample of the French working population of employees. We used sample weights for all the statistical analyses so that our results could be extrapolated to the population. We examined a large set of employment variables, and various detailed levels of the classifications for occupation and economic activity of the company were used. The exploration of such a long list of detailed employment variables was very seldom in the literature, especially within one single study. We studied gender differences and tested interactions related to gender. No difference was observed between men and women for the prevalence of exposure to job insecurity, but some associations differed between genders. Indeed, the associations of occupation, economic activity of the company, and private/public sector and job insecurity were not the same for both genders. We explored each employment variable in association with job insecurity using prevalence and 95% CI, as well as all these variables simultaneously using multivariate models, although only little differences between models were found. We studied job insecurity as the fear of job loss which may be considered unspecific about the likelihood of being unemployed [18]. This was why we also studied this item of job insecurity in combination with another item related to the difficulty of finding equivalent job. The prevalence of exposure was lower than for job insecurity alone, but the associations with employment variables were very similar, which underlined that the particularly exposed groups were the same.

### Comparison with the literature

The comparison with the literature was done in three steps following the retained classification of the variables: covariates, employment variables including job and company characteristics.

The prevalence of job insecurity found in this study was consistent with previous results in France. A previous study using a nationally representative sample of the French working population in 2006 found a prevalence of exposure to job insecurity of 22% and 23% among women and men respectively, without any gender difference [39]. Our results related to gender were also in agreement with the international literature including reviews/overviews [1,10,17] or other studies [18,20–23,25–27,30] showing no or almost no difference between genders or country-specific associations between gender and job insecurity [28,31]. As suggested by Naswall and De Witte [31], the responsibility for the household income might be more important than gender per se. Lower age was found to be associated with job insecurity in our study. In bivariate association, age groups lower than 50 were associated with a higher prevalence of exposure, but this association was attenuated a little in multivariate models. This was explained by the association of age with job seniority (younger employees are more likely to have lower seniority) and with temporary/permanent work contract (younger employees are more likely to have temporary work contract). The literature provided discordant results on age, as some studies showed higher prevalences of exposure among younger groups [1,21], among middle-age groups [18,20,29,30], among older groups [18,26,28,30,31] or no significant association [10,22,23,25,27]. It should be mentioned that reviews/overviews concluded to no association with age for the most recent review [10], an association between lower age and job insecurity [1] or no consensus [16]. Studies suggested that middle-age groups may be more likely to be exposed to job insecurity due to family responsibility and the heavy impact of potential job loss on their income. Nevertheless, the literature did not always take job seniority and/or work contract into account when studying age in association with job insecurity. Similarly, in our study, the bivariate association showing a higher prevalence of exposure to job insecurity among lower educational level groups disappeared after adjustment for occupation. Indeed, educational level and occupation are strongly associated. Most previous studies, including a review, showed that low educational levels were associated with a higher prevalence of job insecurity [16,20,21,23,26,28,31], in agreement with our bivariate results. However, a review showed no association between educational level and job insecurity [1] and two studies reported a higher prevalence of job insecurity among those with high educational levels [29] or country-specific associations [30]. To sum up, among the three studied individual variables (covariates), only lower age was associated with job insecurity in our study.

In our study, we found that a number of employment variables, as defined by job characteristics, were associated with job insecurity. The most important one was temporary/permanent work contract: temporary workers were more likely to be exposed to job insecurity. This result, which was highly expected, was in line with four reviews/overviews [1,10,16,17] and other previous studies [18,22,24–27,29–32]. Indeed, temporary work contract is defined by a fixed-term contract leading to objective threat to job continuation. In our study, occupation was also an important variable associated with job insecurity. Echoing the results for educational level, the lower the occupational level, the higher the prevalence of job insecurity. Indeed, we found that there was a social gradient in job insecurity, from managers/professionals to blue collar workers, and that unskilled blue collar, in particular industrial, workers had the highest prevalence of exposure to job insecurity. These low-skilled occupations might be more concerned by organisational changes, reorganisation, restructuring, etc. and consequently more exposed to job insecurity. They might also have lower employability and lower opportunities to find another job in case of job loss. This was in agreement with four reviews/overviews [1,10,16,17] and other studies [20–23,29,31], although such a social gradient was not observed in the UK [18] and in Finland [25]. Job seniority was associated with job insecurity in our study, and employees with lower seniority had a higher prevalence of job insecurity. However, this result was slightly attenuated after adjustment for age. The studies exploring job seniority in association with job insecurity were rare, and a review and another study reported a non-significant association [10,23] and two studies showed a U-shape association [26,27]. Interestingly, our results contrast with one of the conclusions of Böckerman’s analysis [23] that long-term attachment to the same company does not reduce job insecurity in Europe. This could be some support for the classic argument of Maurice et al. [40] that French firms are distinctive in terms of the strength of the internal labour market. Full/part time work was not associated with job insecurity in our study, which was in agreement with three reviews and another study [1,10,16,18]. Some studies reported country-specific associations between full/part time work and job insecurity [30,31] or a higher prevalence of job insecurity with full time work [23,26] or with part time work [27,32]. To sum up, the two major employment variables related to job characteristics were temporary work contract and occupation in association with job insecurity in our study.

Amongst the employment variables related to company characteristics, the most important variable was public/private sector, employees working in the private sector having a higher prevalence of job insecurity. This finding was consistent with previous results from the literature [20,22,25,26,30]. One study, however, reported a higher prevalence of job insecurity in the public sector in the UK in 2012 [18], which could be explained by cost-cutting programmes introduced in the public sector to reduce deficits following the 2008 crisis. In our study, economic activity of the company was associated with job insecurity. Our results showed that employees working in the manufacturing sector for both genders, and in the construction sector for men, had the highest prevalence of job insecurity, and that most types of manufactures were particularly exposed. Rare available studies displayed similar results [20,23,25,26,31]. Another study concluded to country-specific associations between sector of activity and job insecurity [30]. We found no association between company size and job insecurity after adjustment for public/private sector. Some seldom studies also found no association [26,27] or country-specific associations [30] or an association between large companies and job insecurity [23]. To sum up, the two major employment variables related to company characteristics were the private sector and economic activity of the company in association with job insecurity in our study.

To our knowledge, only one JEM for job insecurity was constructed previously in Australia using occupation as job title and displayed moderate validity [41]. In addition, a protocol was published recently to present a forthcoming JEM for the US [42]. As the Australian JEM, this US JEM will use occupation as job title, and will take gender and time into account. Our results suggested that JEMs for job insecurity may be relevant as we clearly identified employment variables that were associated with this exposure and that can serve as job title variables. Our study also underlined that occupation may not be the only useful job title variable, and that other employment variables may be important: temporary/permanent work contract, public/private sector, and economic activity of the company. Gender, age and/or job seniority should be taken into account too in the construction of such a JEM. As we showed in a previous publication, constructing JEMs using more than one job title variable is possible methodologically and may be highly suitable for psychosocial work exposures such as job insecurity [19]. Indeed, the use of segmentation methods, for example, can allow the identification of groups that are homogeneous for the studied exposure (i.e. job insecurity), and that can be defined by the combination of several job title variables (i.e. employment variables).

## Conclusions

To conclude, our study provided more information about the employment determinants of job insecurity in the national French working population of employees. Most of our results align well with previously published results for other countries, suggesting that there may not be large differences between France and other countries on this topic. Our results may be useful in a preventive point of view to orient intervention/prevention measures towards specific highly exposed groups. Indeed, job insecurity, as measured by a subjective perception, can also be considered as a good marker to identify vulnerable groups defined by objective job and company characteristics. As stated by De Witte et al. [17], “perceived job insecurity is a subjective reflection of the objective labour market position (and opportunities) of a specific worker”. Our study also suggested that the construction of JEMs for job insecurity may be possible and such JEMs might be a useful tool in the absence of individual exposure data for occupational health studies.

## Supporting information

S1 Table Distribution of job insecurity, age, educational level, and employment variables among the study sample and among men and women separately. (DOCX)

S2 Table Age, educational level, and employment variables in association with job insecurity among the study sample, and among men and women separately: Results from robust Poisson regression models. (DOCX)

S3 Table Age, educational level, and employment variables in association with job insecurity among the study sample, and among men and women separately: Results from robust Poisson regression models. (DOCX)

S4 Table Age, educational level, and employment variables in association with job insecurity among the study sample, and among men and women separately: Results of forward stepwise robust Poisson regression models. (DOCX)

S5 Table Age, educational level, and employment variables in association with job insecurity among the study sample, and among men and women separately: Results of forward stepwise robust Poisson regression models. (DOCX)
