# Supplementary material for: Exploring the employment determinants of job insecurity in the French working population: Evidence from national survey data
Source: PLoS One. 2023 Jun 14;18(6):e0287229. doi: 10.1371/journal.pone.0287229 (PMC10266674; doi:10.1371/journal.pone.0287229)
Supplement: S1 Table — (DOCX) [file pone.0287229.s001.docx]

Supplementary Table S1. Distribution of job insecurity, age, educational level, and employment variables among the study sample and among men and women separately

|  | All (N=28,293)  n (w%) | Men (N=12,283)  n (w%) | Women (N=16,010)  n (w%) | P-value |
| --- | --- | --- | --- | --- |
| **Job insecurity** |  |  |  | 0.314 |
| No | 21,826 (74.8%) | 9,418 (74.3%) | 12,408 (75.2%) |  |
| Yes | 6,467 (25.2%) | 2,865 (25.7%) | 3,602 (24.8%) |  |
| **Age (years)** |  |  |  | 0.007 |
| <30 | 3,657 (18.9%) | 1,694 (19.7%) | 1,963 (18.0%) |  |
| [30-40[ | 7,017 (24.9%) | 3,095 (25.9%) | 3,922 (24.0%) |  |
| [40-50[ | 8,868 (29.0%) | 3,823 (28.3%) | 5,045 (29.7%) |  |
| >=50 | 8,751 (27.2%) | 3,671 (26.2%) | 5,080 (28.3%) |  |
| **Educational level** |  |  |  | <0.001 |
| None | 2,377 (7.8%) | 1,251 (8.6%) | 1,126 (6.9%) |  |
| <A-level | 9,664 (35.0%) | 4,658 (39.2%) | 5,006 (30.8%) |  |
| A-level | 5,021 (17.7%) | 2,065 (17.0%) | 2,956 (18.5%) |  |
| University | 11,195 (39.5%) | 4,290 (35.2%) | 6,905 (43.8%) |  |
| **Occupation (4 groups)** |  |  |  | <0.001 |
| Professionals/managers | 4,720 (19.2%) | 2,591 (22.6%) | 2,129 (15.7%) |  |
| Associate professionals/technicians | 8,441 (26.2%) | 3,345 (25.4%) | 5,096 (27.1%) |  |
| Clerks/service workers | 9,809 (31.1%) | 2,135 (14.5%) | 7,674 (47.9%) |  |
| Blue collar workers | 5,304 (23.5%) | 4,199 (37.5%) | 1,105 (9.4%) |  |
| **Occupation (14 groups)** |  |  |  | <0.001 |
| Professionals working partially as self-employed | 101 (0.3%) | 28 (0.1%) | 73 (0.4%) |  |
| Public service, teaching, science, and cultural professionals | 2,463 (7.5%) | 1,149 (7.4%) | 1,314 (7.6%) |  |
| Business, administration, and engineering professionals | 2,152 (11.4%) | 1,412 (15.1%) | 740 (7.6%) |  |
| Teaching, health, and public service associate professionals | 4,813 (10.6%) | 1,066 (5.6%) | 3,747 (15.6%) |  |
| Business and administration associate professionals | 1,748 (7.9%) | 697 (6.6%) | 1,051 (9.3%) |  |
| Technicians | 1,261 (5.2%) | 1,041 (8.8%) | 220 (1.6%) |  |
| Foremen | 605 (2.5%) | 532 (4.4%) | 73 (0.6%) |  |
| Public service clerks and workers | 5,359 (11.9%) | 1,491 (8.1%) | 3,868 (15.8%) |  |
| Clerks | 1,575 (6.8%) | 216 (1.9%) | 1,359 (11.7%) |  |
| Sales workers | 1,051 (4.6%) | 236 (2.2%) | 815 (7.0%) |  |
| Personal service workers | 1,820 (7.9%) | 190 (2.4%) | 1,630 (13.4%) |  |
| Skilled blue collar workers | 3,544 (15.5%) | 3,038 (27.0%) | 506 (3.9%) |  |
| Unskilled blue collar workers | 1,517 (6.9%) | 983 (8.9%) | 534 (4.9%) |  |
| Agricultural workers | 242 (1.1%) | 177 (1.6%) | 65 (0.5%) |  |
| **Occupation (25 groups)** |  |  |  | <0.001 |
| Professionals working partially as self-employed | 101 (0.3%) | 28 (0.1%) | 73 (0.4%) |  |
| Public service professionals | 838 (2.0%) | 411 (2.1%) | 427 (2.0%) |  |
| Teaching and science professionals | 1,402 (4.0%) | 636 (3.6%) | 766 (4.4%) |  |
| Information and cultural professionals | 222 (1.5%) | 102 (1.7%) | 120 (1.2%) |  |
| Business and administration professionals | 1,174 (6.2%) | 631 (6.6%) | 543 (5.7%) |  |
| Engineering professionals | 974 (5.2%) | 778 (8.5%) | 196 (1.9%) |  |
| Primary, secondary, and vocational education teachers | 1,167 (3.4%) | 348 (2.4%) | 819 (4.5%) |  |
| Health and social work associate professionals | 2,628 (5.4%) | 404 (2.0%) | 2,224 (8.8%) |  |
| Clergy | 9 (0.0%) | 7 (0.0%) | 2 (0.0%) |  |
| Public service associate professionals | 1,009 (1.8%) | 307 (1.2%) | 702 (2.3%) |  |
| Business and administration associate professionals | 1,748 (7.9%) | 697 (6.6%) | 1,051 (9.3%) |  |
| Technicians | 1,261 (5.2%) | 1,041 (8.8%) | 220 (1.6%) |  |
| Foremen | 605 (2.5%) | 532 (4.4%) | 73 (0.6%) |  |
| Public service clerks and personal care workers | 4,667 (9.7%) | 891 (4.1%) | 3,776 (15.3%) |  |
| Protective services workers | 692 (2.2%) | 600 (4.0%) | 92 (0.5%) |  |
| Clerks | 1,575 (6.8%) | 216 (1.9%) | 1,359 (11.7%) |  |
| Sales workers | 1,051 (4.6%) | 236 (2.2%) | 815 (7.0%) |  |
| Personal service workers | 1,820 (7.9%) | 190 (2.4%) | 1,630 (13.4%) |  |
| Skilled industrial workers | 1,107 (5.1%) | 904 (8.4%) | 203 (1.8%) |  |
| Skilled craft workers | 1,450 (5.8%) | 1,254 (10.3%) | 196 (1.2%) |  |
| Drivers | 561 (2.5%) | 516 (4.5%) | 45 (0.4%) |  |
| Skilled handling, storage and transport workers | 422 (2.1%) | 361 (3.7%) | 61 (0.5%) |  |
| Unskilled industrial workers | 820 (3.8%) | 545 (5.2%) | 275 (2.4%) |  |
| Unskilled craft workers | 696 (3.1%) | 438 (3.7%) | 258 (2.5%) |  |
| Agricultural workers | 242 (1.1%) | 177 (1.6%) | 65 (0.5%) |  |
| **Economic activity (4 groups)** |  |  |  | <0.001 |
| Agriculture | 270 (1.1%) | 186 (1.5%) | 84 (0.6%) |  |
| Manufacturing | 3,443 (15.7%) | 2,407 (22.8%) | 1,036 (8.6%) |  |
| Construction | 1,297 (6.3%) | 1,144 (11.2%) | 153 (1.5%) |  |
| Services | 23,132 (76.9%) | 8,468 (64.5%) | 14,664 (89.3%) |  |
| **Economic activity (17 groups)** |  |  |  | <0.001 |
| Agriculture, forestry and fishing | 270 (1.1%) | 186 (1.5%) | 84 (0.6%) |  |
| Manufacture of food products, beverages, and tobacco products | 624 (2.5%) | 343 (2.9%) | 281 (2.1%) |  |
| Manufacture of coke and refined petroleum products | 28 (0.3%) | 24 (0.5%) | 4 (0.0%) |  |
| Manufacture of electrical, electronic and computer products, and machinery | 301 (2.1%) | 224 (3.1%) | 77 (1.0%) |  |
| Manufacture of transport equipment | 434 (1.9%) | 336 (3.0%) | 98 (0.8%) |  |
| Manufacture of other industrial products | 1,612 (7.0%) | 1,125 (9.9%) | 487 (4.1%) |  |
| Mining and quarrying, energy and water supply, waste management and remediation activities | 444 (2.0%) | 355 (3.3%) | 89 (0.6%) |  |
| Construction | 1,297 (6.3%) | 1,144 (11.2%) | 153 (1.5%) |  |
| Wholesale and retail trade, and repair of motor vehicles and motorcycles | 2,378 (12.1%) | 1,142 (12.2%) | 1,236 (12.1%) |  |
| Transportation and storage | 1,147 (5.7%) | 780 (7.9%) | 367 (3.5%) |  |
| Accommodation and food service activities | 664 (3.4%) | 287 (3.3%) | 377 (3.5%) |  |
| Information and communication | 601 (3.0%) | 395 (4.0%) | 206 (2.0%) |  |
| Financial and insurance activities | 721 (3.7%) | 279 (3.0%) | 442 (4.4%) |  |
| Real estate activities | 223 (1.1%) | 105 (1.0%) | 118 (1.2%) |  |
| Scientific and technical activities, and administrative and support service activities | 1,647 (8.9%) | 798 (9.1%) | 849 (8.6%) |  |
| Public administration, education, human health and social work activities | 13,824 (32.1%) | 4,203 (20.9%) | 9,621 (43.3%) |  |
| Other service activities | 1,927 (6.8%) | 479 (3.2%) | 1,448 (10.5%) |  |
| **Economic activity (38 groups)** |  |  |  | <0.001 |
| Agriculture, forestry and fishing | 270 (1.1%) | 186 (1.5%) | 84 (0.6%) |  |
| Mining and quarrying | 34 (0.2%) | 31 (0.3%) | 3 (0.0%) |  |
| Manufacture of food products, beverages, and tobacco products | 624 (2.5%) | 343 (2.9%) | 281 (2.1%) |  |
| Manufacture of textiles, wearing apparel, leather products, and footwear | 116 (0.4%) | 41 (0.3%) | 75 (0.4%) |  |
| Manufacture of wood and paper, and printing | 186 (0.7%) | 130 (1.1%) | 56 (0.4%) |  |
| Manufacture of coke and refined petroleum products | 28 (0.3%) | 24 (0.5%) | 4 (0.0%) |  |
| Manufacture of chemicals and chemical products | 165 (0.7%) | 115 (1.1%) | 50 (0.4%) |  |
| Manufacture of basic pharmaceutical products and pharmaceutical preparations | 143 (0.8%) | 63 (0.6%) | 80 (0.9%) |  |
| Manufacture of rubber and plastic products, and other non-metallic mineral products. | 291 (1.3%) | 209 (1.9%) | 82 (0.7%) |  |
| Manufacture of basic metals and fabricated metal products, except machinery and equipment | 473 (2.0%) | 387 (3.3%) | 86 (0.7%) |  |
| Manufacture of computer, electronic, and optical products | 86 (0.6%) | 53 (0.9%) | 33 (0.3%) |  |
| Manufacture of electrical equipment | 51 (0.3%) | 31 (0.3%) | 20 (0.2%) |  |
| Manufacture of machinery and equipment n.e.c. | 164 (1.2%) | 140 (1.9%) | 24 (0.4%) |  |
| Manufacture of transport equipement | 434 (1.9%) | 336 (3.0%) | 98 (0.8%) |  |
| Other manufacturing activities, and repair and installation of machinery and equipment | 238 (1.1%) | 180 (1.7%) | 58 (0.6%) |  |
| Electricity, gas, steam and air conditioning supply | 197 (1.1%) | 152 (1.8%) | 45 (0.4%) |  |
| Water supply, sewerage, waste management and remediation activities | 213 (0.7%) | 172 (1.2%) | 41 (0.2%) |  |
| Construction | 1,297 (6.3%) | 1,144 (11.2%) | 153 (1.5%) |  |
| Wholesale and retail trade, and repair of motor vehicles and motorcycles | 2,378 (12.1%) | 1,142 (12.2%) | 1,236 (12.1%) |  |
| Transportation and storage | 1,147 (5.7%) | 780 (7.9%) | 367 (3.5%) |  |
| Accommodation and food service activities | 664 (3.4%) | 287 (3.3%) | 377 (3.5%) |  |
| Publishing, programming and broadcasting activities | 163 (1.2%) | 91 (1.4%) | 72 (0.9%) |  |
| Telecommunications | 161 (0.6%) | 106 (0.7%) | 55 (0.4%) |  |
| Computer programming, consultancy and related activities, and information service activities | 277 (1.3%) | 198 (1.9%) | 79 (0.7%) |  |
| Financial and insurance activities | 721 (3.7%) | 279 (3.0%) | 442 (4.4%) |  |
| Real estate activities | 223 (1.1%) | 105 (1.0%) | 118 (1.2%) |  |
| Legal and accounting, management consultancy, architectural and engineering, and technical testing and analysis activities | 471 (2.8%) | 197 (2.6%) | 274 (3.0%) |  |
| Scientific research and development | 238 (1.1%) | 127 (1.0%) | 111 (1.1%) |  |
| Other scientific and technical activities | 190 (1.1%) | 85 (1.1%) | 105 (1.2%) |  |
| Administrative and support service activities | 748 (3.9%) | 389 (4.5%) | 359 (3.4%) |  |
| Public administration | 4,110 (9.1%) | 2,004 (9.4%) | 2,106 (8.8%) |  |
| Education | 3,221 (8.8%) | 957 (5.7%) | 2,264 (12.0%) |  |
| Human health activities | 4,772 (7.8%) | 969 (3.5%) | 3,803 (12.1%) |  |
| Residential care activities, and social work activities without accommodation | 1,721 (6.4%) | 273 (2.3%) | 1,448 (10.4%) |  |
| Arts, entertainment, and recreation activities | 382 (1.3%) | 168 (1.3%) | 214 (1.3%) |  |
| Other service activities | 536 (1.6%) | 162 (0.9%) | 374 (2.2%) |  |
| Activities of households | 966 (3.9%) | 129 (0.8%) | 837 (6.9%) |  |
| Activities of extraterritorial organisations and bodies | 43 (0.1%) | 20 (0.1%) | 23 (0.1%) |  |
| **Public/private sector** |  |  |  | <0.001 |
| Public | 11,919 (23.7%) | 4,175 (18.5%) | 7,744 (29.1%) |  |
| Private | 16,374 (76.3%) | 8,108 (81.5%) | 8,266 (70.9%) |  |
| **Company size** |  |  |  | <0.001 |
| 1-49 | 5,733 (27.3%) | 2,576 (25.3%) | 3,157 (29.4%) |  |
| 50-499 | 3,461 (14.6%) | 1,726 (16.1%) | 1,735 (13.1%) |  |
| 500 or more | 17,841 (58.0%) | 7,404 (58.5%) | 10,437 (57.5%) |  |
| **Permanent/temporary work contract** |  |  |  | 0.340 |
| Permanent | 25,135 (86.3%) | 10,976 (86.7%) | 14,159 (85.9%) |  |
| Temporary | 3,139 (13.7%) | 1,303 (13.3%) | 1,836 (14.1%) |  |
| **Full/part time work** |  |  |  | <0.001 |
| Full time | 22,690 (81.3%) | 11,528 (94.6%) | 11,162 (67.9%) |  |
| Part time | 5,374 (18.7%) | 687 (5.4%) | 4,687 (32.1%) |  |
| **Seniority (years)** |  |  |  | 0.280 |
| <=1 | 2,229 (11.7%) | 1,099 (12.4%) | 1,130 (11.1%) |  |
| ]1-5] | 5,381 (24.4%) | 2,388 (24.4%) | 2,993 (24.5%) |  |
| ]5-10] | 5,029 (17.7%) | 2,117 (17.4%) | 2,912 (18.0%) |  |
| >10 | 15,616 (46.1%) | 6,667 (45.8%) | 8,949 (46.5%) |  |

n (w%): unweighted number (weighted %)

P-value for the comparison between genders (Rao-Scott Chi-2 test)
