# Supplementary material for: Exploring the employment determinants of job insecurity in the French working population: Evidence from national survey data
Source: PLoS One. 2023 Jun 14;18(6):e0287229. doi: 10.1371/journal.pone.0287229 (PMC10266674; doi:10.1371/journal.pone.0287229)
Supplement: S2 Table — (DOCX) [file pone.0287229.s002.docx]

Supplementary Table S2. Age, educational level, and employment variables in association with job insecurity among the study sample, and among men and women separately: results from robust Poisson regression models

|  | All  (N=26,610) | | | Men  (N=11,549) | | | Women  (N=15,061) | | |
| --- | --- | --- | --- | --- | --- | --- | --- | --- | --- |
|  | PR | 95% CI | P-value | PR | 95% CI | P-value | PR | 95% CI | P-value |
| **Gender** |  |  | 0.092 |  |  |  |  |  |  |
| Men | 1 |  |  |  |  |  |  |  |  |
| Women | 1.08 | 0.99; 1.17 |  |  |  |  |  |  |  |
| **Age (years)** |  |  | <0.001 |  |  | <0.001 |  |  | 0.007 |
| <30 | **0.85*** | **0.74; 0.98** |  | 0.90 | 0.74; 1.10 |  | **0.83*** | **0.68; 1.00** |  |
| [30-40[ | **1.15*** | **1.03; 1.28** |  | **1.23**** | **1.05; 1.44** |  | 1.09 | 0.93; 1.27 |  |
| [40-50[ | **1.15**** | **1.04; 1.28** |  | **1.29***** | **1.12; 1.49** |  | 1.05 | 0.91; 1.21 |  |
| >=50 | 1 |  |  | 1 |  |  | 1 |  |  |
| **Educational level** |  |  | 0.647 |  |  | 0.592 |  |  | 0.585 |
| None | 1.09 | 0.94; 1.26 |  | 1.05 | 0.86; 1.28 |  | 1.11 | 0.89; 1.38 |  |
| < A-level | 1.03 | 0.92; 1.15 |  | 0.95 | 0.81; 1.12 |  | 1.11 | 0.95; 1.30 |  |
| A-level | 1.05 | 0.94; 1.18 |  | 1.01 | 0.85; 1.20 |  | 1.09 | 0.94; 1.26 |  |
| University | 1 |  |  | 1 |  |  | 1 |  |  |
| **Occupation (4 groups)** |  |  | 0.008 |  |  | 0.004 |  |  | 0.209 |
| Managers/professionals | 1 |  |  | 1 |  |  | 1 |  |  |
| Associate professionals/technicians | **1.17*** | **1.03; 1.34** |  | 1.16 | 0.97; 1.40 |  | **1.20*** | **1.01; 1.44** |  |
| Clerks/service workers | **1.16*** | **1.01; 1.35** |  | 1.25 | 1.00; 1.58 |  | 1.10 | 0.91; 1.34 |  |
| Blue collar workers | **1.30***** | **1.11; 1.51** |  | **1.44***** | **1.17; 1.76** |  | 1.08 | 0.85; 1.37 |  |
| **Economic activity (4 groups)** |  |  | <0.001 |  |  | 0.069 |  |  | <0.001 |
| Agriculture | **0.50**** | **0.33; 0.76** |  | **0.58*** | **0.35; 0.96** |  | **0.38**** | **0.19; 0.75** |  |
| Manufacturing | **1.16**** | **1.04; 1.28** |  | 1.10 | 0.97; 1.25 |  | **1.29**** | **1.09; 1.53** |  |
| Construction | 1.07 | 0.92; 1.24 |  | 1.03 | 0.88; 1.22 |  | 1.23 | 0.78; 1.94 |  |
| Services | 1 |  |  | 1 |  |  | 1 |  |  |
| **Public/private sector** |  |  | <0.001 |  |  | <0.001 |  |  | <0.001 |
| Public | 1 |  |  | 1 |  |  | 1 |  |  |
| Private | **1.73***** | **1.57; 1.91** |  | **1.95***** | **1.67; 2.29** |  | **1.60***** | **1.41; 1.83** |  |
| **Company size** |  |  | 0.801 |  |  | 0.393 |  |  | 0.362 |
| 1-49 | 1.02 | 0.92; 1.14 |  | 0.95 | 0.82; 1.10 |  | 1.10 | 0.95; 1.28 |  |
| 50-499 | 1.04 | 0.93; 1.15 |  | 1.06 | 0.92; 1.23 |  | 1.01 | 0.86; 1.19 |  |
| 500 or more | 1 |  |  | 1 |  |  | 1 |  |  |
| **Permanent/temporary work contract** |  |  | <0.001 |  |  | <0.001 |  |  | <0.001 |
| Permanent | 1 |  |  | 1 |  |  | 1 |  |  |
| Temporary | **2.30***** | **2.07; 2.54** |  | **2.11***** | **1.80; 2.48** |  | **2.47***** | **2.17; 2.81** |  |
| **Part/full time work** |  |  | 0.783 |  |  | 0.804 |  |  | 0.815 |
| Full time | 1 |  |  | 1 |  |  | 1 |  |  |
| Part time | 1.01 | 0.92; 1.11 |  | 1.02 | 0.85; 1.24 |  | 1.01 | 0.91; 1.13 |  |
| **Seniority (years)** |  |  | 0.042 |  |  | 0.241 |  |  | 0.165 |
| <=1 | 1.08 | 0.94; 1.25 |  | 1.10 | 0.90; 1.36 |  | 1.06 | 0.87; 1.30 |  |
| ]1-5] | **1.12*** | **1.01; 1.26** |  | 1.07 | 0.91; 1.25 |  | **1.16*** | **1.00; 1.35** |  |
| ]5-10] | **1.15**** | **1.04; 1.27** |  | **1.16*** | **1.01; 1.34** |  | 1.13 | 0.97; 1.31 |  |
| >10 | 1 |  |  | 1 |  |  | 1 |  |  |

Occupation and economic activity were studied using the two variables with 4 groups

Poisson regression models with robust variance estimation using weighted data

PR: prevalence rate, CI : confidence interval

All variables were included in the models simultaneously

* p<0.05, **p<0.01, ***<0.001
