# Supplementary material for: Exploring the employment determinants of job insecurity in the French working population: Evidence from national survey data
Source: PLoS One. 2023 Jun 14;18(6):e0287229. doi: 10.1371/journal.pone.0287229 (PMC10266674; doi:10.1371/journal.pone.0287229)
Supplement: S3 Table — (DOCX) [file pone.0287229.s003.docx]

Supplementary Table S3. Age, educational level, and employment variables in association with job insecurity among the study sample, and among men and women separately: results from robust Poisson regression models

|  | All  (N=26,587) | | | Men  (N=11,535) | | | Women  (N=15,052) | | |
| --- | --- | --- | --- | --- | --- | --- | --- | --- | --- |
|  | PR | 95% CI | P-value | PR | 95% CI | P-value | PR | 95% CI | P-value |
| **Gender** |  |  | 0.027 |  |  |  |  |  |  |
| Men | 1 |  |  |  |  |  |  |  |  |
| Women | **1.10*** | **1.01; 1.21** |  |  |  |  |  |  |  |
| **Age (years)** |  |  | <0.001 |  |  | <0.001 |  |  | 0.006 |
| <30 | **0.84*** | **0.74; 0.97** |  | 0.89 | 0.74; 1.08 |  | **0.82*** | **0.68; 0.98** |  |
| [30-40[ | **1.14*** | **1.02; 1.28** |  | **1.23**** | **1.05; 1.44** |  | 1.07 | 0.92; 1.25 |  |
| [40-50[ | **1.15**** | **1.05; 1.28** |  | **1.30***** | **1.13; 1.50** |  | 1.05 | 0.92; 1.20 |  |
| >=50 | 1 |  |  | 1 |  |  | 1 |  |  |
| **Educational level** |  |  | 0.397 |  |  | 0.473 |  |  | 0.770 |
| None | 1.13 | 0.98; 1.31 |  | 1.12 | 0.92; 1.36 |  | 1.10 | 0.88; 1.37 |  |
| < A-level | 1.04 | 0.93; 1.17 |  | 0.99 | 0.84; 1.16 |  | 1.09 | 0.93; 1.27 |  |
| A-level | 1.05 | 0.93; 1.17 |  | 1.03 | 0.86; 1.22 |  | 1.05 | 0.91; 1.23 |  |
| University | 1 |  |  | 1 |  |  | 1 |  |  |
| **Occupation (14 groups)** |  |  | 0.202 |  |  | 0.111 |  |  | 0.289 |
| Professionals working partially as self-employed | 0.88 | 0.44; 1.77 |  | 0.94 | 0.33; 2.72 |  | 0.94 | 0.40; 2.22 |  |
| Public service, teaching, science, and cultural professionals | 1 |  |  | 1 |  |  | 1 |  |  |
| Business, administration, and engineering professionals | 1.10 | 0.85; 1.43 |  | 0.93 | 0.65; 1.33 |  | 1.34 | 0.93; 1.94 |  |
| Teaching, health, and public service associate professionals | **1.35**** | **1.09; 1.66** |  | 1.28 | 0.90; 1.81 |  | **1.38*** | **1.04; 1.83** |  |
| Business and administration associate professionals | 1.28 | 0.99; 1.66 |  | 1.15 | 0.79; 1.67 |  | **1.45*** | **1.02; 2.06** |  |
| Technicians | 1.23 | 0.94; 1.60 |  | 1.06 | 0.75; 1.50 |  | **1.80**** | **1.17; 2.77** |  |
| Foremen | 1.06 | 0.78; 1.45 |  | 1.00 | 0.67; 1.48 |  | 0.90 | 0.46; 1.76 |  |
| Public service clerks and workers | 1.22 | 0.99; 1.52 |  | 1.19 | 0.86; 1.66 |  | 1.21 | 0.91; 1.60 |  |
| Clerks | 1.26 | 0.96; 1.65 |  | 1.34 | 0.89; 2.01 |  | 1.35 | 0.94; 1.94 |  |
| Sales workers | 1.30 | 0.97; 1.74 |  | 1.34 | 0.84; 2.13 |  | 1.34 | 0.90; 2.00 |  |
| Personal service workers | 1.27 | 0.97; 1.67 |  | 0.94 | 0.51; 1.71 |  | 1.37 | 0.97; 1.93 |  |
| Skilled blue collar workers | **1.35*** | **1.06; 1.72** |  | 1.31 | 0.94; 1.82 |  | 1.22 | 0.83; 1.79 |  |
| Unskilled blue collar workers | 1.26 | 0.98; 1.62 |  | 1.28 | 0.90; 1.80 |  | 1.24 | 0.85; 1.81 |  |
| Agricultural workers | 1.17 | 0.71; 1.93 |  | 0.75 | 0.38; 1.50 |  | **1.75*** | **1.00; 3.06** |  |
| **Economic activity (17 groups)** |  |  | <0.001 |  |  | <0.001 |  |  | <0.001 |
| Agriculture, forestry and fishing | 0.72 | 0.43; 1.19 |  | 1.23 | 0.68; 2.25 |  | **0.35**** | **0.17; 0.73** |  |
| Manufacture of food products, beverages, and tobacco products | 0.96 | 0.75; 1.22 |  | 1.03 | 0.72; 1.47 |  | 0.90 | 0.64; 1.27 |  |
| Manufacture of coke and refined petroleum products | 0.65 | 0.21; 1.97 |  | 0.49 | 0.13; 1.92 |  | 1.48 | 0.28; 7.85 |  |
| Manufacture of electrical, electronic and computer products, and machinery | **1.61**** | **1.20; 2.16** |  | **1.47*** | **1.00; 2.15** |  | **2.19***** | **1.44; 3.33** |  |
| Manufacture of transport equipment | **1.94***** | **1.53; 2.46** |  | **2.04***** | **1.49; 2.79** |  | **1.85**** | **1.21; 2.82** |  |
| Manufacture of other industrial products | **1.66***** | **1.41; 1.97** |  | **1.72***** | **1.33; 2.22** |  | **1.61***** | **1.26; 2.05** |  |
| Mining and quarrying, energy and water supply, waste management and remediation activities | 1.01 | 0.74; 1.37 |  | 1.07 | 0.73; 1.55 |  | 0.93 | 0.54; 1.61 |  |
| Construction | **1.39***** | **1.15; 1.69** |  | **1.43**** | **1.10; 1.87** |  | 1.36 | 0.84; 2.21 |  |
| Wholesale and retail trade, and repair of motor vehicles and motorcycles | **1.36***** | **1.14; 1.62** |  | **1.34*** | **1.01; 1.78** |  | **1.37**** | **1.09; 1.72** |  |
| Transportation and storage | **1.43***** | **1.19; 1.71** |  | **1.47**** | **1.13; 1.92** |  | **1.39*** | **1.05; 1.85** |  |
| Accommodation and food service activities | **1.36*** | **1.05; 1.77** |  | **1.63*** | **1.07; 2.47** |  | 1.27 | 0.89; 1.80 |  |
| Information and communication | 1.27 | 0.96; 1.69 |  | 1.39 | 0.94; 2.07 |  | 1.15 | 0.79; 1.66 |  |
| Financial and insurance activities | 0.89 | 0.66; 1.20 |  | 1.17 | 0.73; 1.88 |  | 0.73 | 0.50; 1.05 |  |
| Real estate activities | 1.41 | 0.88; 2.24 |  | 1.41 | 0.83; 2.41 |  | 1.39 | 0.74; 2.61 |  |
| Scientific and technical activities, and administrative and support service activities | **1.40***** | **1.18; 1.67** |  | **1.67***** | **1.27; 2.19** |  | 1.14 | 0.91; 1.43 |  |
| Public administration, education, human health and social work activities | 1 |  |  | 1 |  |  | 1 |  |  |
| Other service activities | 1.19 | 0.99; 1.42 |  | 1.12 | 0.79; 1.58 |  | 1.13 | 0.93; 1.38 |  |
| **Public/private sector** |  |  | <0.001 |  |  | <0.001 |  |  | 0.013 |
| Public | 1 |  |  | 1 |  |  | 1 |  |  |
| Private | **1.42***** | **1.22; 1.66** |  | **1.54***** | **1.21; 1.98** |  | **1.31*** | **1.06; 1.62** |  |
| **Company size** |  |  | 0.574 |  |  | 0.352 |  |  | 0.498 |
| 1-49 | 1.02 | 0.91; 1.14 |  | 0.97 | 0.84; 1.13 |  | 1.10 | 0.94; 1.28 |  |
| 50-499 | 1.06 | 0.95; 1.19 |  | 1.09 | 0.94; 1.26 |  | 1.03 | 0.88; 1.21 |  |
| 500 or more | 1 |  |  | 1 |  |  | 1 |  |  |
| **Permanent/temporary work contract** |  |  | <0.001 |  |  | <0.001 |  |  | <0.001 |
| Permanent | 1 |  |  | 1 |  |  | 1 |  |  |
| Temporary | **2.35***** | **2.12; 2.60** |  | **2.17***** | **1.85; 2.55** |  | **2.50***** | **2.19; 2.84** |  |
| **Part/full time work** |  |  | 0.619 |  |  | 0.621 |  |  | 0.667 |
| Full time | 1 |  |  | 1 |  |  | 1 |  |  |
| Part time | 1.02 | 0.93; 1.13 |  | 1.05 | 0.87; 1.27 |  | 1.02 | 0.92; 1.14 |  |
| **Seniority (years)** |  |  | 0.034 |  |  | 0.240 |  |  | 0.119 |
| <=1 | 1.06 | 0.92; 1.23 |  | 1.07 | 0.88; 1.31 |  | 1.05 | 0.86; 1.28 |  |
| ]1-5] | **1.13*** | **1.01; 1.26** |  | 1.06 | 0.91; 1.24 |  | **1.17*** | **1.00; 1.35** |  |
| ]5-10] | **1.15**** | **1.03; 1.27** |  | **1.16*** | **1.01; 1.33** |  | 1.13 | 0.98; 1.31 |  |
| >10 | 1 |  |  | 1 |  |  | 1 |  |  |

Occupation and economic activity were studied using the two variables with 14 and 17 groups, respectively

Poisson regression models with robust variance estimation using weighted data

PR: prevalence rate, CI : confidence interval

All variables were included in the models simultaneously

* p<0.05, **p<0.01, ***<0.001
