# Supplementary material for: Exploring the employment determinants of job insecurity in the French working population: Evidence from national survey data
Source: PLoS One. 2023 Jun 14;18(6):e0287229. doi: 10.1371/journal.pone.0287229 (PMC10266674; doi:10.1371/journal.pone.0287229)
Supplement: S4 Table — (DOCX) [file pone.0287229.s004.docx]

Supplementary Table S4. Age, educational level, and employment variables in association with job insecurity among the study sample, and among men and women separately: results of forward stepwise robust Poisson regression models

|  | All  (N=28,075) | | | Men  (N=12,190) | | | Women  (N=15,922) | | |
| --- | --- | --- | --- | --- | --- | --- | --- | --- | --- |
|  | PR | 95% CI | P-value | PR | 95% CI | P-value | PR | 95% CI | P-value |
| **Gender†** |  |  | 0.094 |  |  |  |  |  |  |
| Men | 1 |  |  |  |  |  |  |  |  |
| Women | 1.07 | 0.99; 1.16 |  |  |  |  |  |  |  |
| **Permanent/temporary work contract** |  |  | <0.001 |  |  | <0.001 |  |  | <0.001 |
| Permanent | 1 |  |  | 1 |  |  | 1 |  |  |
| Temporary | **2.18***** | **1.98; 2.39** |  | **2.12***** | **1.87; 2.41** |  | **2.36***** | **2.12; 2.62** |  |
| **Public/private sector** |  |  | <0.001 |  |  | <0.001 |  |  | <0.001 |
| Public | 1 |  |  | 1 |  |  | 1 |  |  |
| Private | **1.70***** | **1.57; 1.85** |  | **1.94***** | **1.69; 2.23** |  | **1.65***** | **1.50; 1.82** |  |
| **Age (years)** |  |  | <0.001 |  |  | <0.001 |  |  | 0.061 |
| <30 | 0.89 | 0.78; 1.01 |  | 0.99 | 0.83; 1.18 |  | 0.91 | 0.77; 1.07 |  |
| [30-40[ | **1.16**** | **1.05; 1.29** |  | **1.31***** | **1.13; 1.51** |  | 1.10 | 0.96; 1.27 |  |
| [40-50[ | **1.16**** | **1.06; 1.28** |  | **1.32***** | **1.16; 1.52** |  | 1.06 | 0.93; 1.22 |  |
| >=50 | 1 |  |  | 1 |  |  | 1 |  |  |
| **Occupation (4 groups)** |  |  | <0.001 |  |  | <0.001 |  |  |  |
| Managers/professionals | 1 |  |  | 1 |  |  |  |  |  |
| Associate professionals/technicians | **1.18**** | **1.04; 1.34** |  | 1.15 | 0.97; 1.38 |  |  |  |  |
| Clerks/service workers | **1.19**** | **1.05; 1.35** |  | 1.22 | 0.99; 1.51 |  |  |  |  |
| Blue collar workers | **1.38***** | **1.22; 1.56** |  | **1.43***** | **1.21; 1.68** |  |  |  |  |
| **Economic activity (4 groups)** |  |  | <0.001 |  |  | 0.004 |  |  | <0.001 |
| Agriculture | **0.48***** | **0.32; 0.73** |  | **0.51**** | **0.31; 0.84** |  | **0.43*** | **0.22; 0.83** |  |
| Manufacturing | **1.20***** | **1.09; 1.31** |  | **1.14*** | **1.02; 1.29** |  | **1.30***** | **1.12; 1.50** |  |
| Construction | 1.07 | 0.93; 1.24 |  | 1.04 | 0.89; 1.21 |  | 1.19 | 0.73; 1.94 |  |
| Services | 1 |  |  | 1 |  |  | 1 |  |  |
| **Seniority (years)** |  |  | 0.031 |  |  |  |  |  |  |
| <=1 | 1.09 | 0.96; 1.25 |  |  |  |  |  |  |  |
| ]1-5] | **1.13*** | **1.02; 1.26** |  |  |  |  |  |  |  |
| ]5-10] | **1.14**** | **1.03; 1.26** |  |  |  |  |  |  |  |
| >10 | 1 |  |  |  |  |  |  |  |  |

Occupation and economic activity were studied using the two variables with 4 groups

Poisson regression models with robust variance estimation using weighted data

PR: prevalence rate, CI : confidence interval

† Forced variable in the model

Variables presented in the order of selection for all.

The order of selection for men was: Public/private sector, Permanent/temporary work contract, Age (years), Occupation (4 groups), Economic activity (4 groups)

The order of selection for women was: Permanent/temporary work contract, Public/private sector, Economic activity (4 groups), Age (years)
