# Supplementary material for: Exploring the employment determinants of job insecurity in the French working population: Evidence from national survey data
Source: PLoS One. 2023 Jun 14;18(6):e0287229. doi: 10.1371/journal.pone.0287229 (PMC10266674; doi:10.1371/journal.pone.0287229)
Supplement: S5 Table — (DOCX) [file pone.0287229.s005.docx]

Supplementary Table S5. Age, educational level, and employment variables in association with job insecurity among the study sample, and among men and women separately: results of forward stepwise robust Poisson regression models

|  | All  (28,065) | | | Men  (12,176) | | | Women  (15,922) | | |
| --- | --- | --- | --- | --- | --- | --- | --- | --- | --- |
|  | PR | 95% CI | P-value | PR | 95% CI | P-value | PR | 95% CI | P-value |
| **Gender†** |  |  | 0.005 |  |  |  |  |  |  |
| Men | 1 |  |  |  |  |  |  |  |  |
| Women | **1.12**** | **1.03; 1.20** |  |  |  |  |  |  |  |
| **Permanent/temporary work contract** |  |  | <0.001 |  |  | <0.001 |  |  | <0.001 |
| Permanent | 1 |  |  | 1 |  |  | 1 |  |  |
| Temporary | **2.25***** | **2.05; 2.47** |  | **2.18***** | **1.93; 2.46** |  | **2.36***** | **2.13; 2.62** |  |
| **Public/private sector** |  |  | <0.001 |  |  | <0.001 |  |  | <0.001 |
| Public | 1.00 | — |  | 1.00 | — |  | 1.00 | — |  |
| Private | **1.44***** | **1.28; 1.62** |  | **1.58***** | **1.26; 1.98** |  | **1.43***** | **1.25; 1.63** |  |
| **Economic activity (17 groups)** |  |  | <0.001 |  |  | <0.001 |  |  | <0.001 |
| Agriculture, forestry and fishing | **0.65*** | **0.42; 0.99** |  | 1.14 | 0.63; 2.08 |  | **0.51*** | **0.26; 0.98** |  |
| Manufacture of food products, beverages, and tobacco products | 1.05 | 0.86; 1.30 |  | 1.04 | 0.75; 1.45 |  | 1.08 | 0.83; 1.42 |  |
| Manufacture of coke and refined petroleum products | **2.03**** | **1.21; 3.41** |  | **2.12**** | **1.24; 3.64** |  | 1.47 | 0.28; 7.81 |  |
| Manufacture of electrical, electronic and computer products, and machinery | **1.66***** | **1.27; 2.17** |  | **1.53*** | **1.07; 2.18** |  | **2.17***** | **1.44; 3.28** |  |
| Manufacture of transport equipment | **1.96***** | **1.58; 2.43** |  | **1.98***** | **1.49; 2.63** |  | **1.78**** | **1.25; 2.53** |  |
| Manufacture of other industrial products | **1.71***** | **1.48; 1.98** |  | **1.71***** | **1.35; 2.18** |  | **1.65***** | **1.34; 2.03** |  |
| Mining and quarrying, energy and water supply, waste management and remediation activities | 0.97 | 0.73; 1.30 |  | 1.02 | 0.72; 1.47 |  | 0.95 | 0.57; 1.58 |  |
| Construction | **1.42***** | **1.19; 1.68** |  | **1.41**** | **1.10; 1.81** |  | 1.41 | 0.86; 2.31 |  |
| Wholesale and retail trade, and repair of motor vehicles and motorcycles | **1.38***** | **1.19; 1.60** |  | **1.33*** | **1.02; 1.75** |  | **1.42***** | **1.19; 1.69** |  |
| Transportation and storage | **1.46***** | **1.24; 1.72** |  | **1.45**** | **1.13; 1.87** |  | **1.38*** | **1.07; 1.78** |  |
| Accommodation and food service activities | **1.31*** | **1.04; 1.66** |  | **1.52*** | **1.02; 2.28** |  | 1.35 | 0.99; 1.84 |  |
| Information and communication | 1.26 | 0.97; 1.62 |  | 1.45 | 1.00; 2.10 |  | 1.14 | 0.83; 1.59 |  |
| Financial and insurance activities | 0.88 | 0.67; 1.16 |  | 1.13 | 0.72; 1.77 |  | 0.75 | 0.54; 1.05 |  |
| Real estate activities | 1.27 | 0.80; 2.03 |  | 1.36 | 0.82; 2.25 |  | 1.30 | 0.64; 2.62 |  |
| Scientific and technical activities, and administrative and support service activities | **1.39***** | **1.19; 1.63** |  | **1.58***** | **1.22; 2.06** |  | **1.27*** | **1.04; 1.54** |  |
| Public administration, education, human health and social work activities | 1 |  |  | 1 |  |  | 1 |  |  |
| Other service activities | 1.12 | 0.96; 1.30 |  | 1.05 | 0.76; 1.46 |  | 1.18 | 1.00; 1.40 |  |
| **Age (years)** |  |  | <0.001 |  |  | <0.001 |  |  | 0.047 |
| <30 | 0.90 | 0.79; 1.02 |  | 0.96 | 0.81; 1.14 |  | 0.90 | 0.77; 1.05 |  |
| [30-40[ | **1.17**** | **1.05; 1.30** |  | **1.29***** | **1.12; 1.49** |  | 1.10 | 0.96; 1.26 |  |
| [40-50[ | **1.17**** | **1.07; 1.29** |  | **1.33***** | **1.16; 1.52** |  | 1.07 | 0.94; 1.21 |  |
| >=50 | 1 |  |  | 1 |  |  | 1 |  |  |
| **Educational level** |  |  | 0.009 |  |  |  |  |  |  |
| None | **1.21**** | **1.07; 1.36** |  |  |  |  |  |  |  |
| < A-level | **1.13**** | **1.03; 1.23** |  |  |  |  |  |  |  |
| A-level | **1.12*** | **1.01; 1.24** |  |  |  |  |  |  |  |
| University | 1 |  |  |  |  |  |  |  |  |
| **Seniority (years)** |  |  | 0.010 |  |  |  |  |  |  |
| <=1 | 1.10 | 0.96; 1.25 |  |  |  |  |  |  |  |
| ]1-5] | **1.15**** | **1.04; 1.28** |  |  |  |  |  |  |  |
| ]5-10] | **1.16**** | **1.05; 1.28** |  |  |  |  |  |  |  |
| >10 | 1 |  |  |  |  |  |  |  |  |
| **Occupation (14 groups)** |  |  |  |  |  | 0.005 |  |  |  |
| Professionals working partially as self-employed |  |  |  | 0.91 | 0.31; 2.65 |  |  |  |  |
| Public service, teaching, science, and cultural professionals |  |  |  | 1 |  |  |  |  |  |
| Business, administration, and engineering professionals |  |  |  | 0.92 | 0.65; 1.30 |  |  |  |  |
| Teaching, health, and public service associate professionals |  |  |  | 1.24 | 0.88; 1.74 |  |  |  |  |
| Business and administration associate professionals |  |  |  | 1.14 | 0.79; 1.63 |  |  |  |  |
| Technicians |  |  |  | 1.05 | 0.76; 1.47 |  |  |  |  |
| Foremen |  |  |  | 1.05 | 0.72; 1.53 |  |  |  |  |
| Public service clerks and workers |  |  |  | 1.21 | 0.89; 1.64 |  |  |  |  |
| Clerks |  |  |  | 1.38 | 0.94; 2.02 |  |  |  |  |
| Sales workers |  |  |  | 1.32 | 0.85; 2.04 |  |  |  |  |
| Personal service workers |  |  |  | 0.89 | 0.50; 1.59 |  |  |  |  |
| Skilled blue collar workers |  |  |  | 1.34 | 0.99; 1.80 |  |  |  |  |
| Unskilled blue collar workers |  |  |  | 1.33 | 0.98; 1.82 |  |  |  |  |
| Agricultural workers |  |  |  | 0.72 | 0.37; 1.40 |  |  |  |  |

Occupation and economic activity were studied using the two variables with 14 and 17 groups, respectively

Poisson regression models with robust variance estimation using weighted data

PR: prevalence rate, CI : confidence interval

† Forced variable in the model

Variables presented in the order of selection for all and women.

The order of selection for men was: Public/private sector, Permanent/temporary work contract, Age (years), Economic activity (17 groups), Occupation (14 groups),
